# Relapsing localized pagetoid reticulosis despite radiation therapy

**DOI:** 10.1016/j.jdcr.2025.01.010

**Published:** 2025-02-04

**Authors:** Sara Suhl, Tessa M. LeWitt, Alexander Kaminsky, Brigit A. Lapolla, Larisa J. Geskin

**Affiliations:** a Columbia University Vagelos College of Physicians and Surgeons, New York, New York; b Department of Dermatology, Columbia University Irving Medical Center, New York, New York

**Keywords:** case report, CTCL, cutaneous T-cell lymphoma, MF, mycosis fungoides, pagetoid reticulosis, radiation, radiotherapy, recurrent, relapsing, Woringer-Kolopp

## Introduction

Localized pagetoid reticulosis (PR), or Woringer-Kolopp disease, is a variant of mycosis fungoides (MF) that classically presents as a slow-growing solitary psoriasiform or hyperkeratotic patch/plaque on a distal extremity.^1^ There are currently no treatment guidelines or known prognostic biomarkers specifically for PR. PR is defined, in part, by its slow disease course and excellent response to appropriate treatment; however, it can significantly impact quality of life, especially for those with extensive, relapsing, or refractory disease. Here, we discuss a case of PR with multiple relapses following radiation with complete remission (CR).

## Case report

The patient is a 50-year-old woman with diabetes mellitus and a 6-year history of asymptomatic plaques on her plantar and lateral right foot without an identifiable trigger. She was treated empirically for psoriasis with an unknown topical compounded treatment by a podiatrist without improvement. One year later, a dermatologist biopsied a plaque on the right foot, which showed MF, PR type. Her lesions persisted despite several months of topical clobetasol. She transitioned to daily mechlorethamine 0.016% gel with improvement, but had to discontinue treatment due to allergic contact dermatitis. She then presented to our cutaneous T-cell lymphoma specialty clinic.

**Fig 1:**
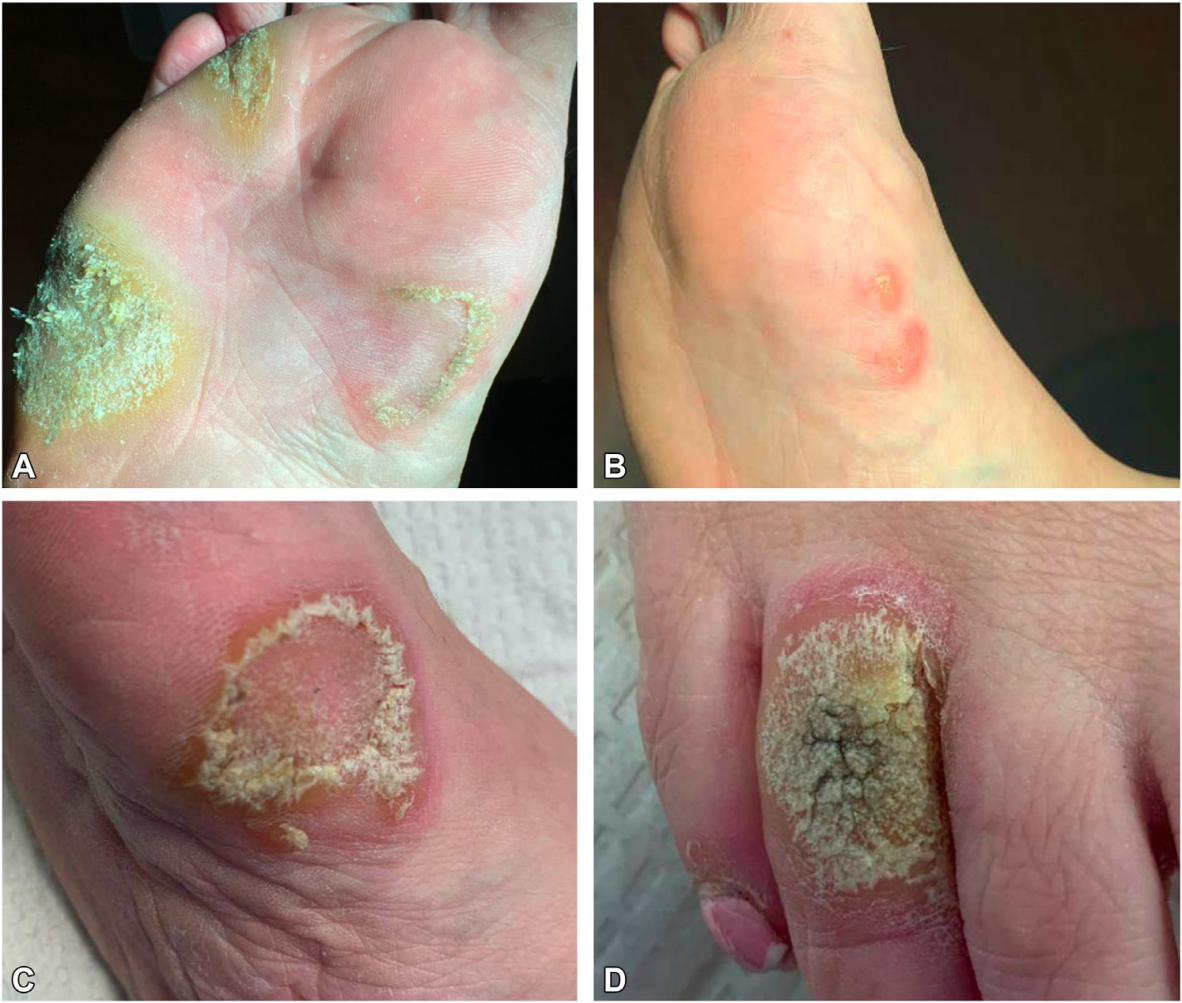
Pagetoid reticulosis of the foot before and after radiation therapy. Right plantar foot at initial presentation to cutaneous oncologist (**A**), new foot lesions on medial instep <4 m after remission from treatment with 2400 cGy radiation (**B**), progression of foot lesion on the medial instep (**C**) and new lesion on the dorsal toe (**D**) after 6 m.

**Fig 2:**
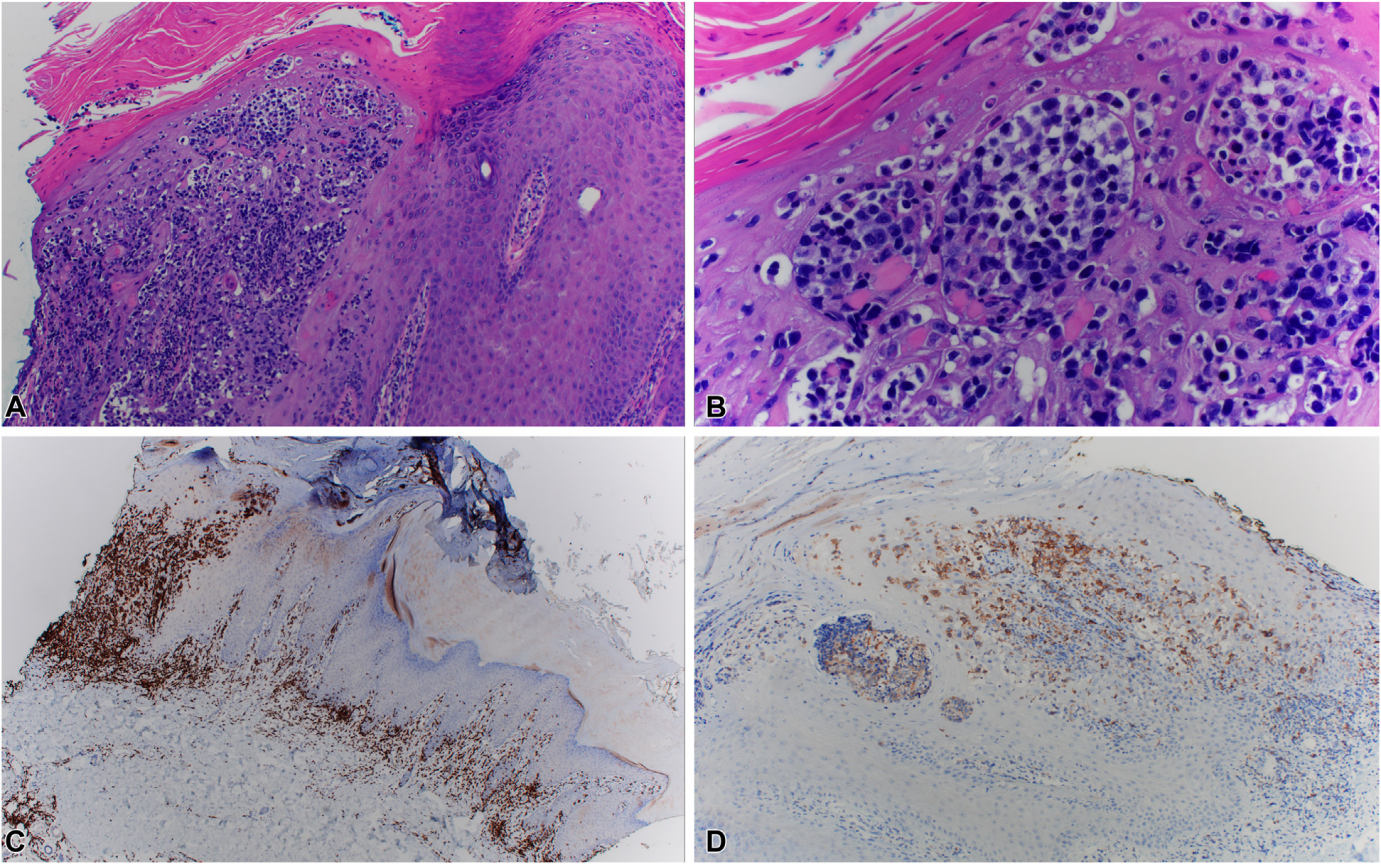
Histologic examination of recurrent pagetoid reticulosis of the foot. H&E stain showing atypical epidermotropic lymphocytic infiltrate with prominent pseudoepitheliomatous changes at 100× (**A**) and 400× magnification (**B**). Abnormal lymphocytes with positive CD3+ (**C**) staining (40× magnification) (**C**) and 30% CD30 positivity (100× magnification) (**D**).

**Fig 3:**
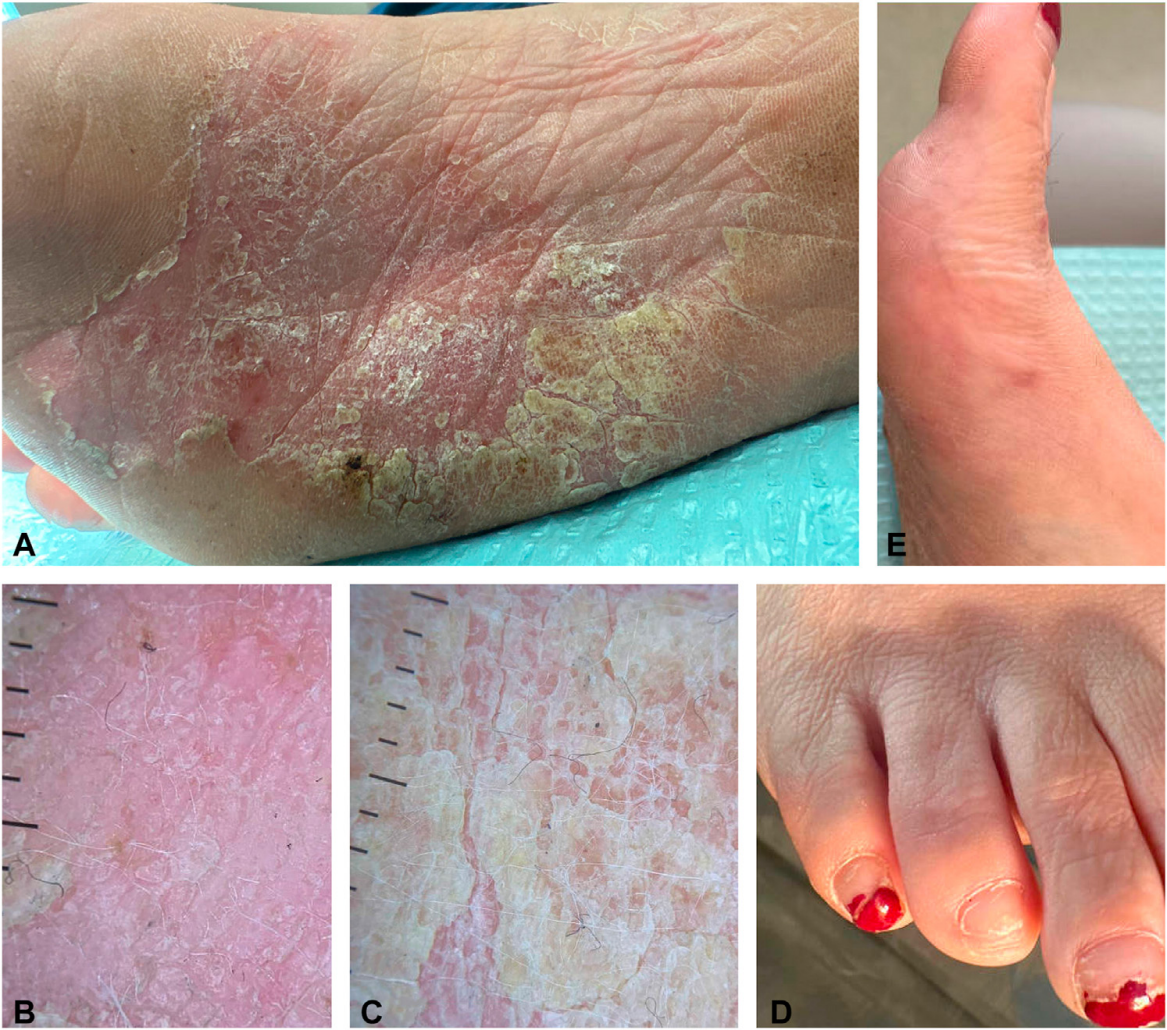
Relapsed pagetoid reticulosis of the foot after radiation therapy. Right plantar foot with new erythematous hyperkeratotic plaque of the sole (**A**), Dermatoscopic images of the new right plantar plaque (**B** and **C**). Dorsal toe (**D**) and medial foot (**E**) and with complete remission following treatment with 2400 cGy radiation.

Physical exam was notable for multiple thick hyperkeratotic plaques on the right plantar foot (Fig 1, *A*). Punch biopsy confirmed CD8+ PR, with histopathology notable for malignant cells in the papillary dermis and lower third of the epidermis (Table I). While a diagnosis of MF Palmaris et Plantaris was considered given dermal involvement, PR was favored due the other clinical (hyperkeratotic plaques, local spread) and immunohistochemical (CD8+, elevated Ki-67, CD30) features more classic for PR. Her persistent plaques were treated with radiation monotherapy (2400 cGy 12 fractions over 3 sessions) with CR. However, after <4 months, she re-presented with 2 erythematous arcuate papules on previously uninvolved areas of the right foot instep, outside the radiation field (Fig 1, *B*). To rule out inflammation secondary to fungal infection, she began topical antifungal and topical steroid use. On follow-up 6 months later, she had disease progression on the right medial instep (Fig 1, *C*), fourth toe (Fig 1, *D*), and interdigital space. Repeat biopsy showed atypical epidermotropic lymphocytic infiltrate with prominent pseudoepitheliomatous change, tagging of cells along the dermal-epidermal junction, frequent microabscesses, intermediate cells, and 30% large cells (Fig 2, *A* and *B*), consistent with MF, PR type with increased large cells. Most lymphocytes were CD4-/CD8+, abnormal lymphocytes were CD3+ (Fig 2, *C*) and CD5+, with aberrant loss of CD7, and 30% CD30 positivity (Fig 2, *D*). Ki67 proliferation index was ∼50% within atypical cells, PD-1 staining was positive in ∼10% of cells, and T-cell receptor-gamma was negative. At a multidisciplinary cutaneous T-cell lymphoma tumor board conference, she was diagnosed with recurrent PR given the CR of lesions treated with radiation and presence of new biopsy-proven lesions in a previously uninvolved area outside the prior radiation field. Given her localized area of involvement, previous allergic contact dermatitis to mechlorethamine, and prior excellent response to radiation, we recommended a second course. She received 2400 cGy in 12 fractions to both the dorsal and medial regions of the right foot, resulting in CR. Three months later, the patient began developing a new red, flaky lesion on the sole of the right foot in an area not previously involved. After 3 months of progressive worsening, she re-presented to the cutaneous oncology team with no evidence of disease in the field of recent radiation treatment and a new, large, erythematous, hyperkeratotic plaque on the right sole, with negative scrape biopsy for fungal infection, consistent with a second relapse (Fig 3).

**Table I:** Comparison between clinical and immunohistologic characteristics of multirelapsing pagetoid reticulosis

	Case report	Tan et al^2^	Haghighi et al^3^
Age/Sex	50/F	68/M	50/M
Duration (y)∗	1	16	3
Location	Foot (several lesions)	Wrist	Foot (several lesions)
Treatment	XRT, XRT	XRT ×6, topical 5-FU	XRT, PUVA, XRT
Outcome	Relapse ×2bg	Relapse ×6	Relapse ×2, then lost to F/U
Follow-up (y)†	5.5	12	8
Large cells	yes	yes	NA
Dermal involvement	yes	yes	NA
CD2	+++	NA	NA
CD3	+++	NA	+++
CD4	-	5% (epidermis), 30% (dermis)	NA
CD5	+++	50% (epidermis), 80% (dermis)	NA
CD7	>90% reduction (epidermis), ∼60% reduction (dermis)	NA	NA
CD8	+++	40% (epidermis), 30% (dermis)	-
CD4/CD8 ratio	<1:10 (epidermis) and <1:3 (dermis)	1:8 (epidermis), 1:1 (dermis)	NA
CD30+	Biopsy 1: 40% (epidermis), 15% (dermis)Biopsy 2: 30%	NA	-
Beta F1	+++	NA	-
Ki67	Biopsy 1: +++ (epidermis), 30% (dermis) Biopsy 2: 50%	NA	NA
PD-1	10%	NA	NA
TOX	+++	NA	NA
Granzyme	+++	NA	NA
EBER	-	NA	NA
TCR-delta	-	NA	NA
TCR-gamma	-	NA	NA

*-*, Negative for expression; *+++*, more than 50% expression; *5-FU*, fluorouracil; *F*, female; *F/U*, follow-up; *M*, male; *NA*, information not available; *PUVA*, 8-methoxypsoralen and Ultraviolet A; *TCR*, T-cell receptor; *XRT*, radiation.

∗ Duration of symptoms prior to diagnosis.

† Length of follow-up from diagnosis.

## Discussion

While PR is a rare subtype of an already rare disease, our patient’s clinical course is unique even among those reported in the literature. A 2023 systematic review of PR (Osto et al) included 84 studies of 143 patients. Recurrence of PR in general was unusual, with the majority (78.6%) of cases achieving CR over 43.4 months average follow-up. Radiation was the most utilized treatment, and 100% of patients treated with radiation monotherapy (*n* = 33) achieved CR without recurrence.^4^ Radiation is often recommended for recurrent or refractory disease; however, our patient relapsed twice following radiation courses. While our patient presented with multiple lesions, the vast majority (85.7%, *n* = 114) present with a singular distinct lesion.^4^

Histologically, PR is characterized by significant epidermotropism typically without dermal involvement, yet our patient’s biopsy also possessed malignant cells within the superficial dermis.^5^ While low CD30 positivity, high Ki67 expression, and presence of large cells have been associated with poorer prognosis in lymphomas, currently there is limited evidence supporting a strong relationship between immunophenotypic profile and prognosis in PR.^6^, ^7^, ^8^, ^9^, ^10^ Thus, we reviewed the literature to identify clinical and histopathologic risk factors for multiple episodes of relapse, finding only one other case of multiple relapses with radiation monotherapy. Tan et al described an 68-year-old male patient treated 6 times with “superficial radiotherapy” over 4 years, each time with “good” response but subsequent relapse. Like our patient, he had dermal involvement, the presence of large cells noted on pathology, and a CD4:CD8 ratio of <1:10 (epidermis) and <1:3 (dermis) (Table I).^2^

Expanding our search, we identified one other case of multiple PR relapses. Haghighi et al reported a male patient with multiple foot lesions who relapsed twice following radiation and 8-methoxypsoralen and ultraviolet A treatments with initial CR. Unlike our patient, his infiltrate was CD8-and CD30- (Table I).^3^ Both cases of multirelapsing PR lack comprehensive information but exemplify the heterogeneity in both PR’s clinical and immunohistochemical characteristics, underscoring a need for more data on PR pathology and outcomes to allow for improved prognostication.

Here, we present and comprehensively characterize the unique case of a woman with multiple PR relapses following radiation, suggesting the potential of a more recurrent variant of this condition traditionally characterized by an indolent course, favorable prognosis, and durable response to radiation therapy. This difficult case highlights the potential role for maintenance therapy and underscores the importance of further investigation into prognostic features and novel therapeutic approaches for patients with recurrent disease.

## Conflicts of interest

Dr Geskin has served as an investigator for and/or received research support from Helsinn Group, J&J, Mallinckrodt, Kyowa Kirin, Soligenix, Innate, Merck, BMS, and Stratpharma; on the speakers’ bureau for Helsinn Group and J&J; and on the scientific advisory board for Helsinn Group, J&J, Mallinckrodt, Sanofi, Regeneron, and Kyowa Kirin. Dr LeWitt and Authors Suhl, Kaminsky, and Lapolla have no conflicts of interest to declare.
